# Development of a Predictive Nomogram for Sepsis in Patients with Urolithiasis-Related Obstructive Pyelonephritis

**DOI:** 10.3390/medicina60071113

**Published:** 2024-07-09

**Authors:** Yi-Chun Tsai, Yu-Hsuan Huang, Kuang-Yu Niu, Yu-Chen Tsai, Chen-Bin Chen, Chieh-Ching Yen

**Affiliations:** 1 Department of Nursing, Chang Gung University of Science and Technology, Taoyuan 333, Taiwan; ycn.tsai@gmail.com; 2 Department of Emergency Medicine, Chang Gung Memorial Hospital, Linkou Branch, Taoyuan 333, Taiwan; purplethink29@gmail.com (Y.-H.H.); peidra.niu@gmail.com (K.-Y.N.); 3 Department of Diagnostic Radiology, Chang Gung Memorial Hospital, Keelung 204, Taiwan; b0105036@cgmh.org.tw; 4 Department of Emergency Medicine, New Taipei Municipal Tucheng Hospital, New Taipei City 236, Taiwan; ans76ers@gmail.com; 5 Institute of Emergency and Critical Care Medicine, National Yang Ming Chiao Tung University, Taipei 112, Taiwan

**Keywords:** uolithiasis-related obstructive pyelonephritis, sepsis, nomogram

## Abstract

*Background and Objectives*: In patients with urolithiasis-related obstructive pyelonephritis (UROP), sepsis represents a critical and concerning complication that can substantially increase the mortality rate. This study aimed to identify the risk factors for sepsis in UROP patients and to develop a predictive nomogram model. *Materials and Methods*: We analyzed data from 148 patients who met the UROP criteria and were admitted to Chang Gung Memorial Hospital between 1 January 2016 and 31 December 2021. The primary outcome evaluated was the incidence of sepsis, as defined by the most recent Sepsis-3 guidelines. To identify potential risk factors for sepsis, we employed the Least Absolute Shrinkage and Selection Operator (LASSO) regression technique. Subsequently, we utilized multivariable logistic regression to construct the predictive model. *Results*: There was a total of 102 non-sepsis cases and 46 sepsis cases. Risk factors for sepsis in multivariable analysis were a history of diabetes mellitus (DM) (OR = 4.24, *p* = 0.007), shock index (SI) (×10^−1^) (OR = 1.55, *p* < 0.001), C-reactive protein (CRP) (mg/dL) (OR = 1.08, *p* = 0.005), and neutrophil to lymphocyte ratio (NLR) (×10) (OR = 1.58, *p* = 0.007). The nomogram exhibited an area under the receiver operating characteristic curve of 0.890 (95% CI 0.830–0.949). *Conclusions*: Our study demonstrated that patients with UROP who have DM, higher SI, higher NLR, and elevated CRP levels are significantly more likely to develop sepsis. These insights may aid in risk stratification, and it is imperative that clinicians promptly initiate treatment for those identified as high risk.

## 1. Introduction

Urinary tract infection (UTI) poses a significant healthcare burden and can lead to severe and life-threatening complications, especially when complicated by underlying urinary tract or systemic diseases such as anatomical abnormalities, diabetes mellitus (DM), steroid use, or chemotherapy [1,2]. Acute pyelonephritis (APN), one of the most common types of UTIs, is broadly categorized into uncomplicated and complicated forms. Complicated APN occurs in patients who have either structural or functional abnormalities in their urinary tract [2]. Among a range of urological conditions associated with complicated APN, urolithiasis is notably one of the most frequent [3]. The obstruction of the urinary tract, often caused by stones, results in elevated intrarenal pelvic pressure, leading to localized kidney lesions and potentially life-threatening systemic inflammatory disorders [3].

Sepsis presents a critical concern in the context of urolithiasis-related obstructive pyelonephritis (UROP) [4]. Approximately 10% of upper UTIs with obstruction progress to urosepsis [5]. Mortality rates vary by gender, with 0.73% in females and 1.65% in males for APN alone [6]. When APN progresses to urosepsis, the mortality rate can dramatically escalate. A previous study reported that 42% of patients with complicated APN-developed bacteremia, leading to urosepsis in 4% of these patients [7]. The overall mortality rate of bacteremic APN patients was found to be 6.7%, but this rate surged to 25.9% among those presenting with septic shock [8]. These findings emphasize the urgency for timely diagnosis and intervention, given that delay often leads to rapid progression with fatal outcomes.

Although some risk factors for sepsis in patients with UROP have been proposed, none of them have been integrated into a predictive model. The nomogram has been accepted as a reliable tool for creating a simple, intuitive graph based on a statistical predictive model, which quantifies the risk of a clinical event. Therefore, this study aims to develop a predictive nomogram for sepsis in patients with UROP.

## 2. Materials and Methods

### 2.1. Study Design and Setting

The present study was approved by the Chang Gung Medical Foundation Institutional Review Board (IRB no. 202301921B0) and was conducted in accordance with the Declaration of Helsinki. The data were from the Department of Emergency Medicine, Chang Gung Memorial Hospital, which is a tertiary referral center with a capacity of 3700 beds, 100,000 annual admissions, and 200,000 annual emergency department (ED) visits in Taiwan. All adult patients who met the inclusion criteria of the study during the period of 1 January 2016 to 31 December 2021 were retrospectively enrolled for analysis.

### 2.2. Patient Selection and Data Collection

Through searching the electronic medical records (EMRs) during the study period, all the UROP patients diagnosed at our hospital were first identified. A diagnosis of UROP was made if the patient met all of the following criteria: (1) More than 5 white blood cells (WBCs) in a centrifuge urinary specimen and related characteristic symptoms such as flank or abdominal pain [9]. (2) Imaging appearance of APN, including affected areas appearing swollen, with lower attenuation, perinephric stranding, in contrast-enhanced or non-contrast computed tomography (CT), or wedge-shaped zones of decreased attenuation in contrast-enhanced CT [10]. (3) CT scan to detect hydronephrosis and ureteral calculi as the cause. We excluded patients under the age of 18, patients with duplicated data, and patients without hospitalization. We excluded patients with symptoms which could be attributed to previous surgical interventions in order to reduce potential confounding factors associated with the prognosis of UROP patients. Additionally, patients who met sepsis criteria at the initial presentation in the ED were also excluded, as the outcome of the present study was the development of sepsis. The selected patients were further reviewed by two physicians (Y.-C.T. and Y.-H.H.) for their inclusion eligibility.

For each identified patient, demographic information, which included age, sex, initial vital signs upon admission, and comorbidities such as hypertension, DM, heart failure, malignancy, prior ureteral calculi, prior urinary tract infection, and prior APN were retrieved. Data regarding initial presentations, laboratory findings, CT report, organisms identified from blood and urine cultures, treatment modalities and hospital length of stay (LOS) were collected.

### 2.3. Outcome Measures

The primary outcome was the development of sepsis during hospitalization. We defined sepsis with Sepsis-3 criteria, using the Sequential Organ Failure Assessment score being higher than two points [11]. Patients who met the sepsis criteria, had vasopressors initiated, and exhibited lactate values exceeding 2 mmol/L, even after adequate fluid resuscitation during their hospital stay, were categorized into the septic shock group. Patients were initially assessed over the first two days following admission to determine urgent treatments, including antibiotic treatment only, percutaneous nephrostomy (PCN), ureteral stent, and ureteroscopy lithotripsy (URSL). These early interventions were confirmed by reviewing imaging findings and surgical records in the EMRs under both ED and inpatient settings. Subsequently, patients were followed up throughout their hospital stay to monitor in-hospital outcomes, including LOS, intensive care unit (ICU) admission, development of sepsis and septic shock.

### 2.4. Model Construction and Statistical Analysis

Patient characteristics, comorbidities, initial presentations, laboratory findings, urinalysis, CT findings, organisms, treatment modalities, and in-hospital outcomes were recorded as numbers (percentages) for categorical variables, and as mean ± standard deviation (SD) for continuous variables. Categorical comparisons between sepsis and non-sepsis groups utilized the chi-square test or Fisher’s exact test based on cell frequencies. For normally distributed continuous variables, independent Student’s *t*-tests were conducted, while skewed variables were analyzed with Mann–Whitney U-tests. We used the Least Absolute Shrinkage and Selection Operator (LASSO) regression with 10-fold cross-validation to identify predictive factors for sepsis risk in patients with UROP [12]. This method, featuring an L1 penalty, aids in addressing multicollinearity and overfitting. We selected the largest lambda within one standard error of the minimum mean squared error (MSE) through cross-validation, and applied multivariable logistic regression with the chosen predictors (*p* < 0.05) to develop the model. Model performance was assessed by discriminative ability (via the area under the receiver operating characteristic curve) and calibration (assessed by a calibration plot and Hosmer–Lemeshow goodness-of-fit test). A well-calibrated model is signified by a *p* value greater than 0.05 in the Hosmer–Lemeshow test. We employed bootstrap resampling for internal validation of our model, fitting regression models to 1000 bootstrap replicates drawn with replacement from the original sample of UROP patients to estimate optimism in model performance [13]. A bias-corrected concordance index (C-index) was utilized to assess discriminative ability. Furthermore, to quantify the clinical usefulness of the developed nomogram, decision curve analysis (DCA) was applied to ascertain the net benefits to patients at different threshold probabilities. All analyses were performed using R Version 4.1.2 (R Foundation for Statistical Computing, Vienna, Austria). We considered a two-sided *p* value of <0.05 as indicative of statistical significance.

## 3. Results

### 3.1. Patient Characteristics

**Figure 1:**
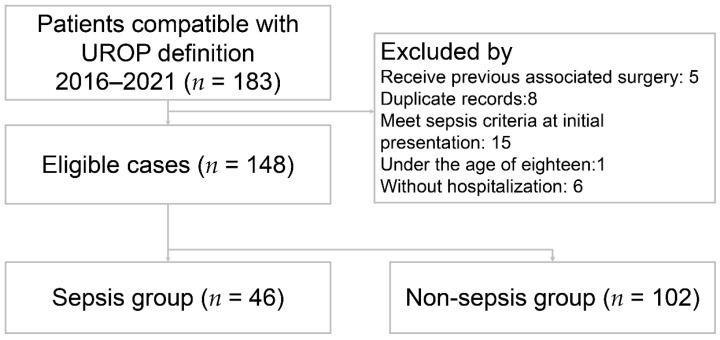
Flow diagram of eligible patients. UROP: urolithiasis-related obstructive pyelonephritis.

A total of 148 patients met the inclusion criteria and were included in the study (Figure 1). The patient characteristics are presented in Table 1. The mean age was 60.8 ± 14.6 years. Fever was present in 58 (39.2%) patients. The percentage of female was 73.6%. Among all the UROP patients, 66 (44.6%) had hypertension, 50 (33.8%) had DM, 56 (37.8%) had prior urolithiasis, 16 (10.8%) had prior UTI, and 12 (8.1%) had prior APN. The majority of initial presentations involved 86 (58.1%) patients with flank or back pain, followed by 21 (14.2%) with abdominal pain, 19 (12.8%) with dysuria, and 11 (7.4%) with hematuria. Based on the Sepsis-3 criteria, 46 (31.1%) patients were enrolled in the sepsis group and the others were in the non-sepsis group. The sepsis group showed significantly lower systolic blood pressure (*p* < 0.001), higher heart rate (*p* = 0.002), and a higher prevalence of comorbidity with DM (*p* = 0.015) in the sepsis group. The laboratory results indicated the following differences between the sepsis and non-sepsis groups: serum white blood cell counts (WBC) were 17.2 ± 10.9 (10^3^/µL) versus 12.8 ± 6.4 (10^3^/µL) (*p* = 0.014); bandemia (defined as band form > 3%) was 26.1% versus 4.1% (*p* < 0.001); C-reactive protein (CRP) levels were 19.9 ± 9.8 (mg/dL) in contrast to 9.9 ± 9.9 (mg/dL) (*p* < 0.001); neutrophil to lymphocyte ratio (NLR) was 23.6 ± 20.3 versus 12.9 ± 10.1 (*p* < 0.001); and positive blood culture rates were 60.9% versus 33.3% (*p* = 0.002).

### 3.2. Microbiology Results

Blood and urine cultures were performed on all 148 patients to identify the causative pathogen (Table 2). Of these, 62 (41.9%) showed evidence of bacteremia, while 79 (53.4%) had positive urine cultures. The most common pathogen found in the blood cultures was *Escherichia coli* (*E. coli*) (*n* = 49, 70.0%), with ESBL (Extended-Spectrum Beta-Lactamase)-producers constituting 30.6% of all *E. coli* isolates. This was followed by *Klebsiella pneumoniae* (*n* = 4, 6.4%) and *Proteus mirabilis* (*n* = 3, 4.8%). The predominant pathogen in urine cultures was *E. coli*, accounting for 63.3% (*n* = 50) of cases. Of these, 30.2% were ESBL-producers. Following this, *Klebsiella pneumoniae* represented 8.5% (*n* = 7), while both *Enterococcus faecalis* and *Proteus mirabilis* each constituted 5.1% (*n* = 4) of the cases, making up the majority of the remaining isolates.

### 3.3. Treatment and Outcomes

Each patient received intravenous antibiotics once UROP diagnosis. In total, 111 (75%) patients received intravenous antibiotics without further procedures, 30 (20.3%) received PCN, 7 (4.7%) received ureteral stent insertion, and 4 (2.7%) underwent URSL within two days. There were no significant differences in treatment modalities across the sepsis and non-sepsis groups. Eleven (7.4%) patients developed septic shock during their ED stay, two (1.4%) were admitted to the ICU, and two (1.4%) died. The mean hospital LOS was 10.6 ± 6.4 days, with the sepsis group significantly longer than the non-sepsis group (13.3 vs. 9.3 days, *p* < 0.001) (Table 3). Patients with septic shock are listed in Table 4.

### 3.4. Variable Selection and Model Construction

**Figure 2:**
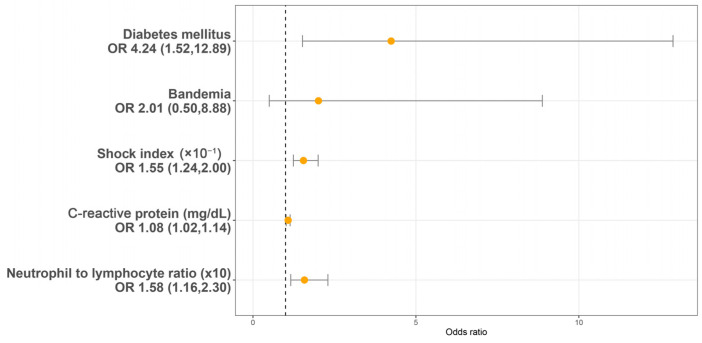
Multivariable logistic regression analysis identifying predictors of sepsis in patients with urolithiasis-related obstructive pyelonephritis. OR: Odds ratio.

Utilizing a LASSO regression model with 10-fold cross-validation, we analyzed variables, including patient characteristics, comorbidities, initial presentations, laboratory findings, urinalysis, and CT findings assessed at the initial ED visit. Following the LASSO regression selection, five variables remained as non-zero coefficients that minimized the overall Lambda and served as the potentially optimal variables for predicting sepsis. These included the shock index (SI), DM, CRP, NLR, and bandemia. Upon incorporating these five variables into a multivariate logistic regression model, four were identified as independently significant predictors of sepsis. These included the SI (×10^−1^) (OR 1.55, 95% CI 1.54–2.00, *p* < 0.001), a history of DM (OR 4.24, 95% CI 1.52–12.89, *p* = 0.007), CRP (mg/dL) (OR 1.08, 95% CI 1.02–1.14, *p* = 0.005), and NLR (×10) (OR = 1.58, 95% CI 1.16–2.30, *p* = 0.007) (Figure 2). Subsequently, based on these significant predictors, we constructed a nomogram.

### 3.5. Performance of the Nomograms and Bootstrap Internal Validation

**Figure 3:**
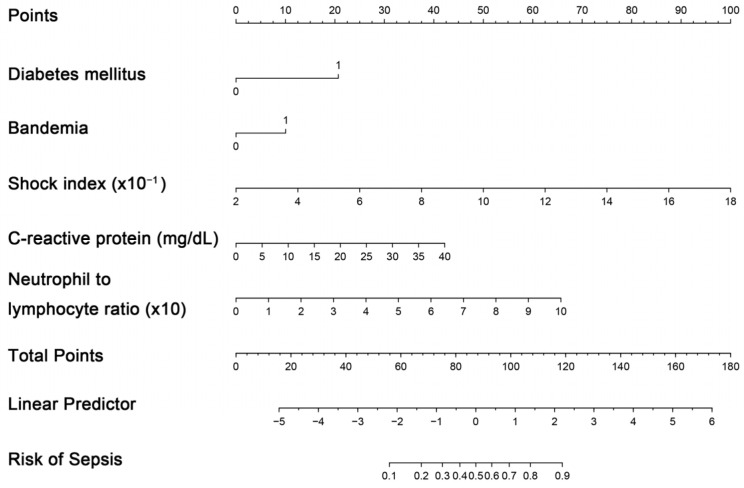
Nomogram for estimating probability of sepsis. Predictor points are identified for each subject variable on the top scale, summed, and the total projected onto the bottom scale to determine the sepsis probability.

**Figure 4:**
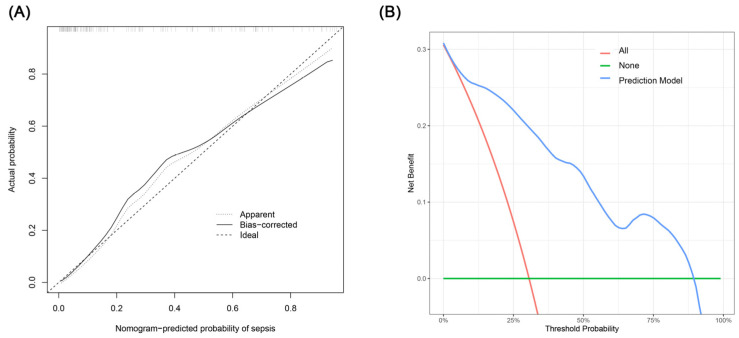
(**A**) Calibration curve for the sepsis nomogram. The x-axis depicts the predicted sepsis risk, while the y-axis shows the actual diagnosed cases of sepsis. The diagonal dotted line illustrates a perfect prediction from an ideal model. The short-dashed line represents the nomogram’s apparent prediction, with the solid line indicating the nomogram’s performance after bias-correction through bootstrapping (B = 1000 repetitions). (**B**) Decision curve analysis for the nomogram. The x-axis displays the threshold probability, while the y-axis quantifies the net benefit. The blue solid line represents the nomogram. The red line assumes that all subjects had sepsis, and the green line assumes that none of the subjects had sepsis.

**Figure 5:**
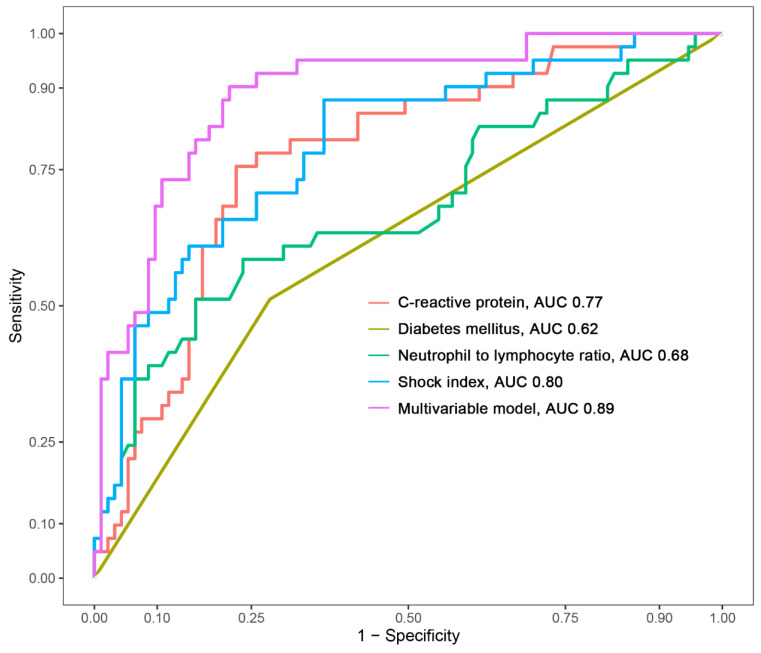
Comparison of the area under the curve (AUC) for the multivariable model and each selected variable.

The creation of a nomogram, depicted in Figure 3, utilized each variable’s value, linking it to a specific score. By summing the scores of the four key variables, we determined the total score for each patient, which was then correlated to the probability of sepsis risk. The nomogram exhibited robust discriminative ability, reflected by an area under the receiver operating characteristic curve (AUC) of 0.890 (95% CI 0.830–0.949). For internal validation, the optimism-corrected concordance index (C-index), based on 1000 bootstrap resamples, was 0.875, indicating substantial reliability. The model displayed effective calibration, as demonstrated by the Hosmer–Lemeshow test (*p* = 0.408). A calibration curve, derived from 1000 bootstrap resamples, demonstrated the close agreement between the predicted and observed probabilities for sepsis (Figure 4A). Utilizing Decision Curve Analysis, we found that the nomogram model delivers a significant net benefit over a wide range of probabilities, notably within the threshold probabilities of approximately 10% to 85% (Figure 4B). Additionally, we presented a comparison of the diagnostic performance of the model with each selected variable in Figure 5.

## 4. Discussion

This retrospective study provides a thorough examination of the clinical characteristics observed in patients with UROP. It also analyzes risk factors for sepsis, including some that have not been previously reported. Our findings indicate that DM, higher SI, higher NLR, and increased levels of CRP are significantly associated with a higher likelihood of sepsis. Based on these selected predictors, we developed a nomogram to predict the risk of sepsis. This nomogram is well-calibrated and offers precise individualized predictions, thereby facilitating more targeted treatment plans. 

Urolithiasis often leads to ureteral obstruction, significantly increasing the likelihood of sepsis as one of its severe complications. It is noteworthy that an estimated 41% of patients experiencing pyelonephritis due to ureteral obstruction from stones or anatomical anomalies progress to sepsis [14]. The severity of this complication is shaped by two pivotal elements: host response and local conditions. In addition to local risk factors such as urolithiasis, obstructive uropathy, congenital uropathy, and neurogenic bladder disorders, UROP patients often exhibit compromised immune systems [4]. This vulnerability is exacerbated in specific high-risk groups, such as the elderly, patients with DM, and those who are immunosuppressed, including organ transplant recipients and patients with AIDS or on corticosteroid therapy [15]. This convergence of host vulnerabilities and local risk factors amplifies the challenges faced in treating this vulnerable population.

Our study found a significant association between DM and the increased risk of developing sepsis in UROP patients. Preclinical studies have indicated that DM interacts with various elements of the innate immune system in vitro, including chemotaxis, phagocytosis, and the activation of neutrophils and macrophages [16]. Animal models of DM further substantiate that hyperglycemia is linked to impaired bacterial clearance, which could contribute to elevated mortality rates among diabetic animals in sepsis experiments [17]. Previous studies have also shown a strong correlation between DM and sepsis in UROP, corroborating our findings [4,18]. In light of this, physicians should be particularly vigilant when treating UROP in diabetic patients. Early intervention through aggressive antibiotic therapy and blood glucose management could be pivotal in mitigating the risk of sepsis in this subgroup.

Our study underscores that a higher shock index, which is not documented in the literature, is a significant predictor for sepsis in UROP patients. Shock index, calculated as the ratio of heart rate to systolic blood pressure, serves as a valuable gauge of hemodynamic stability. As a quick and efficient tool, shock index can alert clinicians to potential underlying issues such as occult bleeding, hemorrhage, or sepsis that might otherwise be overlooked if vital signs, like heart rate and systolic blood pressure, were examined separately [19]. Previous studies have similarly identified high shock index as an independent predictor for severe sepsis and septic shock [20,21]. Given the utility of shock index in assessing systemic hemodynamic stress, physicians should consider incorporating it into initial evaluations to identify UROP patients at elevated risk for sepsis. Early hemodynamic stabilization strategies, such as fluid resuscitation and vasopressor administration, could prove particularly effective in mitigating sepsis risk in this patient population [22].

Our study highlights an association between elevated levels of CRP and the NLR with an increased risk of developing sepsis in patients diagnosed with UROP. CRP is an acute-phase reactant frequently used to assist in diagnosing bacterial infections. It is produced mainly in the liver in response to IL-6, and CRP levels can rise not only during infections but also in various types of inflammation [23]. Similarly, the NLR, calculated by dividing the number of neutrophils by the number of lymphocytes in a blood sample, serves as an effective gauge of systemic inflammation and stress response. As a widely-recognized marker for systemic inflammatory responses, NLR helps to provide insight into the body’s inflammatory state. Specifically, lymphocytes work to counteract non-specific inflammation, while neutrophils play a vital role in responding to non-specific inflammatory triggers and in the secretion of destructive enzymes and inflammatory mediators [24]. Elevated levels of these markers indicate a heightened inflammatory state that may escalate to sepsis. The underlying mechanism appears to involve an overactive and dysregulated inflammatory response, which is a critical factor in the onset and progression of sepsis [25,26,27]. Previous research is consistent with our findings, confirming the role of elevated CRP as a predictor of sepsis [4]. Furthermore, studies have indicated that NLR can predict the need for ureteral catheterization in patients with renal colic and may also serve as a predictor of sepsis in patients undergoing percutaneous nephrolithotomy [28,29]. Clinicians should pay close attention to these inflammatory markers when evaluating these vulnerable patients.

We found that *E. coli* was the most often isolated bacteria from both blood and urine samples, which is consistent with recent studies [30,31]. Importantly, a significant proportion (24.2% in blood culture and 20.3% in urine culture) of these *E. coli* isolates were ESBL-producers, which complicates treatment choices for urosepsis cases. The increased use of third generation cephalosporins and quinolones has led to increasing resistance to these antibiotics [32,33]. Previous studies have raised concerns about relying solely on urine cultures for treatment decisions in patients with obstructive pyelonephritis. These studies found that between 40 and 60% of patients had negative urine cultures, and approximately 80% had negative blood cultures [34]. Our study aligns with these findings, showing a similar rate of negative urine cultures at 46.6% but with a slightly lower rate of negative blood cultures at 58.1%. These results support the importance of incorporating both culture results and antibiotic resistance profiles into a more comprehensive and accurate treatment strategy.

The management of UROP is warranted by urgent decompression, which can be achieved either by PCN or ureteral stent placement, thereby decreasing complications in high-risk patients [35]. One meta-analysis showed no significant difference between the two methods regarding the improvement of septic parameters, quality of life, failure rates, or post-procedural pain [36]. However, managing UROP with PCN in the acute setting might be more advantageous, as it can be performed without spinal anesthesia and provides more effective drainage. This method is particularly suitable for older patients and those considering future percutaneous calculi treatment [37].

### Limitations

This study has several limitations. First, its retrospective design resulted in a certain degree of missing data, and the effects of confounding variables could not be entirely excluded. Traditional risk factors, such as albumin, could not be analyzed because many patients did not have the necessary data. Second, the diagnosis of UROP was established through CT findings, potentially indicating that the clinical severity of the patients included in our study might be more serious compared to those who did not undergo CT examination in the ED. Third, the varied treatment strategies for UROP patients in our tertiary hospital could potentially influence the outcomes. However, the findings might still be applicable to real-world clinical practice. Fourth, the study had a small sample size and we did not perform external validation of our predictive model. As a result, the generalizability of our findings may be limited. Lastly, our study was carried out in Taiwan, a monoethnic country with a predominantly Asian population. As a result, our findings might not fully represent the broader population. Further prospective multi-center studies with larger populations and independent datasets are needed to validate our predictive nomogram and assess its robustness and applicability across different clinical settings.

## 5. Conclusions

In conclusion, patients with UROP who have DM, higher SI, higher NLR, and increased levels of CRP are significantly associated with a higher likelihood of sepsis. The nomogram we developed provides a user-friendly, quantitative tool that integrates multiple clinical variables, which are routinely assessed during initial patient evaluation. By using this tool, clinicians can quickly calculate a risk score that predicts the likelihood of sepsis, facilitating more informed decision-making regarding the need for aggressive treatment strategies or closer monitoring. Further studies with larger numbers of participants will be useful for updating and validating this predictive model.

## Figures and Tables

**Table 1:** Demographics, clinical characteristics and laboratory results between patients with or without sepsis.

Variable	Total (*n* = 148)	Sepsis (*n* = 46)	Non-Sepsis (*n* = 102)	*p* Value
Age (year)	60.8 ± 14.6	62.8 ± 13.6	59.9 ± 15.1	0.268
Male	39 (26.4)	14 (30.4)	25 (24.5)	0.449
SBP (mmHg)	140.9 ± 33.3	122.9 ± 31.7	149.0 ± 30.8	<0.001 *
SBP < 90 (mmHg)	8 (5.4)	7 (15.2)	1 (1.0)	<0.001 *
Heart rate (beats/min)	101.0 ± 20.8	109.2 ± 19.3	97.3 ± 20.5	0.001 *
Heart rate > 100 (beats/min)	72 (48.6)	31 (67.4)	41 (40.2)	0.002 *
Respiratory rate (beats/min)	18.5 ± 2.0	19.2 ± 2.5	18.1 ± 1.7	0.007 *
Shock index	0.8 ± 0.3	0.9 ± 0.3	0.7 ± 0.2	<0.001 *
Fever ^†^	58 (39.2)	21 (45.7)	37 (36.3)	0.279
Initial presentations				
Abdominal pain	21 (14.2)	3 (6.5)	18 (17.6)	0.073
Flank or back pain	86 (58.1)	23 (50.0)	63 (61.8)	0.179
Urinary frequency	1 (0.7)	0 (0.0)	1 (1.0)	0.500
Dysuria	19 (12.8)	8 (17.4)	11 (10.8)	0.266
Hematuria	11 (7.4)	3 (6.5)	8 (7.8)	0.777
Comorbidities				
Hypertension	66 (44.6)	21 (45.7)	45 (44.1)	0.862
Diabetes mellitus	50 (33.8)	22 (47.8)	28 (27.5)	0.015 *
CAD	12 (8.1)	4 (8.7)	8 (7.8)	0.860
CKD	21 (14.2)	6 (13.0)	15 (14.7)	0.789
CHF	4 (2.7)	2 (4.3)	2 (2.0)	0.407
Malignancy	14 (9.5)	5 (10.9)	9 (8.8)	0.694
Prior stroke	4 (2.7)	2 (4.3)	2 (2.0)	0.407
Liver cirrhosis	2 (1.4)	1 (2.2)	1 (1.0)	0.561
Prior urolithiasis	56 (37.8)	12 (25.5)	44 (32.8)	0.351
Prior UTI	16 (10.8)	3 (6.5)	13 (12.7)	0.259
Prior APN	12 (8.1)	3 (6.5)	9 (8.8)	0.635
Blood test ^†^				
White blood count (10^3^/µL)	14.2 ± 8.3	17.2 ± 10.9	12.8 ± 6.4	0.014 *
Bandemia (>3%)	17 (11.5)	12 (26.1)	5 (4.1)	<0.001 *
Hemoglobin (g/dL)	12.2 ± 2.3	11.9 ± 2.4	12.4 ± 2.2	0.161
Platelet (10^3^/µL)	218.4 ± 117.4	193.2 ± 134.7	230.0 ± 107.4	0.078
C-reactive protein (mg/dL)	12.9 ± 10.8	19.9 ± 9.8	9.9 ± 9.9	<0.001 *
Neutrophil to lymphocyte ratio	16.2 ± 14.8	23.6 ± 20.3	12.9 ± 10.1	<0.001 *
Positive blood culture	62 (41.9)	28 (60.9)	34 (33.3)	0.002 *
Urinalysis ^‡^				
Positive urinary nitrite	54 (36.5)	20 (43.5)	34 (33.3)	0.235
WBC (/HPF)	14.2 ± 8.3	17.2 ± 10.9	12.8 ± 6.4	0.014 *
RBC (/HPF)	12.2 ± 2.3	11.9 ± 2.4	12.4 ± 2.2	0.161
Positive urine culture	79 (53.4)	28 (60.9)	51 (50.0)	0.220
CT findings				0.525
Right-side stone	13 (8.8)	6 (13.0)	7 (6.9)	
Left-side stone	16 (10.8)	4 (8.7)	12 (11.8)	
Bilateral stone	6 (4.1)	1 (2.2)	5 (4.9)	
Degree of hydronephrosis				0.239
Grade I–II	97 (65.5)	27 (58.7)	70 (68.6)	
Grade III–IV	51 (34.5)	19 (41.3)	23 (31.4)	
Location of stone				0.514
Renal pelvis or UPJ	41 (27.7)	13 (28.3)	28 (27.5)	
Upper ureter	44 (29.7)	10 (21.7)	34 (33.3)	
Mid ureter	12 (8.1)	5 (10.9)	7 (6.9)	
Lower ureter	16 (10.8)	7 (14.9)	9 (8.8)	
Size of stone (mm) ^‡^	10.0 ± 10.4	9.5 ± 8.0	10.3 ± 11.3	0.668

Count data are expressed as number (percentage) and continuous values are expressed as mean ± SD; SBP: Systolic blood pressure; CAD: Coronary artery disease; CKD: Chronic kidney disease; CHF: Congestive heart failure; UTI: Urinary tract infection; APN: Acute pyelonephritis; WBC: White blood cells; HPF: high-power field; RBC: Red blood cells; CT: Computed tomography; UPJ: Ureteropelvic junction; ^†^ Defined as body temperature > 38 °C; ^‡^ maximal size; * *p* value < 0.05.

**Table 2:** Microbiology results of blood cultures and urine cultures.

Microorganism	*n* ^†^	%
Blood cultures (*n* = 62)		
*Escherichia coli* (ESBL-producer)	49 (15)	70.0 (24.2)
*Klebsiella pneumoniae*	4	6.4
*Proteus mirabilis*	3	4.8
*Enterobacteriaceae*	2	3.2
*Staphylococcus aureus*	1	1.6
*Morganella*	1	1.6
*Moraxella*	1	1.6
*Serratia marcescens*	1	1.6
*Pseudomonas aeruginosa*	1	1.6
Urine cultures (*n* = 79)		
*Escherichia coli (ESBL-producer)*	50 (16)	63.3 (20.3)
*Klebsiella pneumoniae*	7	8.9
*Enterococcus faecalis*	4	5.1
*Proteus mirabilis*	4	5.1
*Yeast*	3	3.8
*Pseudomonas aeruginosa*	3	3.8
*Group B Streptococcus*	2	2.5
*Citrobacter diversus*	2	2.5
*Staphylococcus aureus*	2	2.5
*Morganella*	1	1.3
*Haemophilus*	1	1.3
*Stenotrophomonas maltophilia*	1	1.3

^†^ The total number of microorganisms exceeded the number of patients with positive cultures, as some patients exhibited polymicrobial growth in their cultures.

**Table 3:** Comparison of treatment and in-hospital outcomes in patients with and without sepsis.

Variable	Total (*n* = 148)	Sepsis (*n* = 46)	Non-Sepsis (*n* = 102)	*p* Value
Initial treatment				
Antibiotic treatment only	111 (75.0)	33 (71.7)	78 (76.5)	0.538
PCN	30 (20.3)	12 (26.1)	18 (17.6)	0.237
Ureteral stent	7 (4.7)	3 (6.5)	4 (3.9)	0.490
URSL	4 (2.7)	1 (2.2)	3 (2.9)	0.790
In-hospital outcomes				
Septic shock	11 (7.4)	11 (23.9)	0 (0.0)	<0.001 *
Intensive care unit admission	2 (1.4)	2 (4.3)	0 (0)	0.034 *
Death	2 (1.4)	1 (2.2)	1 (1.0)	0.561
Length of stay	10.6 ± 6.4	13.3 ± 7.1	9.3 ± 5.6	<0.001 *

Count data are expressed as number (percentage) and continuous values are expressed as mean ± SD; PCN: Percutaneous nephrostomy; URSL: Ureteroscopy lithotripsy; * *p* value < 0.05.

**Table 4:** Description of patients with urolithiasis-related obstructive pyelonephritis who developed septic shock.

No.	Age	Gender	Past Medical History	Symptoms	PCN	Operation	Antibiotic Therapy	Length of Stay (Days)	In-Hospital Mortality
1	52	F	PUD	Left flank pain and dysuria	N	Y (ESWL)	D1–D3: Cefuroxime D4–D20: Piperacillin/Tazobactam	20	N
2	74	F	DM, HTN, Breast cancer	Right flank pain	N	Y (URSL + ureteral stent)	D1–D10: Ceftriaxone	10	N
3	43	F	N	Left back pain	N	N	D1–D2: Ceftriaxone D3–D9: Teicoplanin	9	N
4	74	F	DM, HTN, CHF, CKD	Fever	Y	N	D1–D14: Piperacillin/Tazobactam	14	N
5	84	M	HTN, DM	Hypotension	Y	N	D1–D2: Ceftriaxone + Metronidazole D3–D40: Imipenem/Cilastatin	40	Y
6	54	F	HTN, Endometrial cancer	Left flank pain and dysuria	N	N	D1–D11: Ertapenem	11	N
7	66	F	HTN, DM, Cervical cancer	Fever	N	N	D1–D3: Ceftriaxone D4–D20: Imipenem/Cilastatin	20	Y
8	81	F	DM, CKD	Fever and conscious disturbance	N	Y (URSL)	D1–30: Cefoperazone/Sulbactam	30	N
9	70	F	DM	Nausea and vomiting	Y	N	D1–D10: Ceftriaxone	10	N
10	60	M	DM	Fever	N	N	D1–D2: Ertapenem D3–D23: Teicoplanin	23	N
11	70	M	HTN, DM	Fever	N	N	D1–D5: Ceftriaxone D6–D23: Meropenem	23	N

PUD: Peptic ulcer disease; ESWL: Extracorporeal shock wave lithotripsy; DM: Diabetes mellitus; HTN: Hypertension; URSL: Ureteroscopy lithotripsy; CHF: Congestive heart failure; CKD: Chronic kidney disease; Y: Yes; N: No.

## Data Availability

The datasets presented in this article are not readily available because the participants of this study did not give written consent for their data to be shared publicly; thus, due to the sensitive nature of the research, supporting data is not available.
